# Three novel mutations in the *ATP7B* gene of unrelated Vietnamese patients with Wilson disease

**DOI:** 10.1186/s12881-018-0619-4

**Published:** 2018-06-18

**Authors:** Nguyen Thi Mai Huong, Nguyen Thi Kim Lien, Ngo Diem Ngoc, Nguyen Thi Phuong Mai, Nguyen Pham Anh Hoa, Le Thanh Hai, Phan Van Chi, Ta Thanh Van, Tran Van Khanh, Nguyen Huy Hoang

**Affiliations:** 1 Human Genetics Department, Vietnam National Children’s Hospital, Ministry of Health, 18/879 La Thanh str., Dongda, Hanoi, Vietnam; 2 Institute of Genome Research, Vietnam Academy of Science and Technology, 18 - Hoang Quoc Viet str., Caugiay, Hanoi, Vietnam; 3 Hepatology Department, Vietnam National Children’s Hospital, Ministry of Health, 18/879 La Thanh str., Dongda, Hanoi, Vietnam; 4 Vietnam National Children’s Hospital, Ministry of Health, 18/879 La Thanh str., Dongda, Hanoi, Vietnam; 5 Institute of Biotechnology, Vietnam Academy of Science and Technology, 18 - Hoang Quoc Viet str., Caugiay, Hanoi, Vietnam; 6 Hanoi Medical University, Ministry of Education and Training, 1 - Ton That Tung str., Dongda, Hanoi, Vietnam

**Keywords:** Mutation of the *ATP7B* gene, Vietnamese patients, Wilson disease

## Abstract

**Background:**

Wilson disease (OMIM # 277900) is a autosomal recessive disorder characterized by accumulation of copper in liver and brain. The accumulation of copper resulting in oxidative stress and eventually cell death. The disease has an onset in a childhood and result in a significant neurological impairment or require lifelong treatment. Another serious consequence of the disease is the development of liver damage and acute liver failure leading to liver transplant. The disorder is caused by mutations in the *ATP7B* gene, encoding a P-type copper transporting ATPase.

**Case presentation:**

We performed genetic analysis of three unrelated patients from three different Vietnamese families. These patients had clinical features such as numbness of hands and feet, vomiting, insomnia, palsy, liver failure and Kayser–Fleischer (K–F) rings and were diagnosed with Wilson disease in the Human Genetics Department, Vietnam National Children’s Hospital. The entire coding region and adjacent splice sites of *ATP7B* gene were amplified and sequenced by Sanger method. Sequencing data were analyzed and compared with the *ATP7B* gene sequence published in Ensembl (ENSG00000123191) by using BioEdit software to detect mutations.

**Conclusions:**

In this study, five mutations in the *ATP7B* gene were found. Among of these, three mutations were novel: c.750_751insG (p.His251Alafs*19) in exon 2, c.2604delC (p.Pro868Profs*5) in exon 11, and c.3077 T > A (p.Phe1026Tyr) in exon 14. Our results of the mutations associated with Wilson disease might facilitate the development of effective treatment plans.

**Electronic supplementary material:**

The online version of this article (10.1186/s12881-018-0619-4) contains supplementary material, which is available to authorized users.

## Background

Wilson disease (OMIM #277900) is characterized by an accumulation of copper in many organs and tissues [1]. Patients with Wilson disease typically present with low serum Cu and ceruloplasmin concentrations, increased urinary Cu excretion, and/or increased hepatic Cu concentrations, the presence of Kayser-Fleischer rings in the cornea, or neurological/psychiatric symptoms. There is a significantly higher frequency of Wilson disease in East Asian populations than in other populations, ranging from 1:1500 [2] to 1: 3000 [3]. However, clinical manifestations vary between individuals, even within families and in monozygotic twins [4–6]. There is also a wide range in the age of onset, including early-onset hepatic disease in a 3-year-old child and late-onset disease [1] with a mean age of onset of 15.9 years [7]. Indeed, failure diagnosis is the principle cause of death for patients with Wilson disease [8, 9].

Mutations in the *ATP7B* gene were identified as the cause of Wilson disease in 1993 and this gene is still the only one associated with the disorder [10–12]. ATP7B has an essential role in human physiology, particularly in liver and brain function. In the absence of ATP7B function, there is a toxic accumulation of copper in various body tissues, resulting in a wide variety of symptoms, including acute and chronic hepatitis, liver failure, and neurologic dysfunction. *ATP7B* maps on 13q14.3-q21, contains 21 exons and encodes a 1465-amino acids membrane protein [11–13]. ATP7B (copper-transporting ATPase) consists of six metal-binding domains, eight transmembrane segments, and an ATP-binding domain typical of copper ATPases with a P-domain, an N-domain, and an A-domain with the TGE sequence motif [14–16]. Wilson disease is typically caused by homozygous or compound heterozygous mutations in the *ATP7B* gene. Over 776 mutations have been detected in the *ATP7B* gene until now (The Human Gene Mutation Database. http://www.hgmd.cf.ac.uk/ac/index.php). Wilson disease invariably results in severe disability and death, especially for patients with no apparent clinical manifestations who are untreated. The identification of mutations in the *ATP7B* gene is the one of the useful diagnostic tools for diagnosis and treatment orientation for patients with Wilson disease.

In this study, we identified mutations in the *ATP7B* gene of three patients’ families with Wilson disease by sequencing the entire coding region and adjacent splice sites of the *ATP7B* gene. Information about mutations in the *ATP7B* gene will be helpful for efficiently diagnosing Wilson disease and providing early therapeutic intervention for patients.

## Case presentation

### Clinical presentation

#### Patient 1 (WBW140801)

He was an 8-year-old boy who was hospitalized with clinical features such as numbness of hands and feet, vomiting, insomnia, palsy and Kayser–Fleischer (K–F) rings. Biochemical indices of the blood serum revealed 0.0032 mg/dL serum ceruloplasmin (normal is 20–35 mg/dL), 49 μg/dL serum free copper (normal is < 15 μg/dL), 20.1 IU/L ALT (alanine aminotransferase) (normal is < 40 IU/L), 26.82 IU/L AST (aspartate aminotransferase) (normal is < 40 IU/L), and PT (prothrombin time) of 49% (normal is > 70%). In addition, biochemical indices of the urine revealed 580 μg/24 h urinary copper (normal is < 60–100 μg/24 h). The patient had liver failure and severe neurological symptoms. He was diagnosed with Wilson disease in the Human Genetics Department, Vietnam National Children’s Hospital. His parents and his half brother had a normal phenotype. However, we did not collect a sample from his father for genetic analysis.

#### Patient 2 (WBW100604)

She was an 8-year-old girl who was hospitalized with clinical features such as oedema, elevated serum transaminase, liver failure, and Kayser–Fleischer rings. Biochemical indices of the blood serum revealed 0.019 mg/dL serum ceruloplasmin (normal is 20–35 mg/dL), 116.9 IU/L ALT (alanine aminotransferase) (normal is < 40 IU/L), 139.1 IU/L AST (aspartate aminotransferase) (normal is < 40 IU/L), and PT (prothrombin time) of 22% (normal is > 70%). In addition, biochemical indices of the urine revealed 150 μg/24 h urinary copper (normal is < 60–100 μg/24 h). She was diagnosed with Wilson disease in the Human Genetics Department, Vietnam National Children’s Hospital. Her parents and her younger sister had a normal phenotype.

#### Patient 3 (WBW170704)

He was a 10-year-old boy who was hospitalized with clinical features such as oedema, jaundice, acute liver failure, and Kayser–Fleischer rings. Biochemical indices of the blood serum revealed 0.092 mg/dL serum ceruloplasmin (normal is 20–35 mg/dL), 11.6 μg/dL serum free copper (normal is < 15 μg/dL), 55.7 IU/L ALT (alanine aminotransferase) (normal is < 40 IU/L), 129.0 IU/L AST (aspartate aminotransferase) (normal is < 40 IU/L), and PT (prothrombin time) of 17% (normal is > 70%). In addition, biochemical indices of the urine revealed 3639 μg/24 h urinary copper (normal is < 60–100 μg/24 h). He was diagnosed with Wilson disease in the Human Genetics Department, Vietnam National Children’s Hospital. His parents and his younger sister had a normal phenotype.

### Genetic analysis

Genomic DNA was isolated from peripheral blood samples (including sample from patients and their families) using a Qiagen DNA blood mini kit (QIAamp DNA Blood Mini preparation kits, German) following the manufacturer’s guidelines. The DNA concentration was determined using a Thermo Scientific NanoDrop spectrophotometer (Waltham, MA, USA).

All 21 exons and exon-intron boundaries of the *ATP7B* gene were amplified and analysed by direct sequencing. Oligonucleotide primers were synthesized and purchased from IDT (USA) (Additional file 1: Table S1). Fifty nanograms of genomic DNA was subjected to 35 cycles of PCR amplification in a 25 μL volume consisting of 10X PCR buffer (Invitrogen, USA), 10 μM concentration of each primer, 20 mM MgCl_2_, 10 μM dNTPs, and 5 U Taq DNA polymerase (Invitrogen, USA). DNA was denatured at 95 °C for 12 min followed by 35 cycles of denaturation for 1 min at 95 °C, annealing for 1 min at 60–65 °C, and extension for 1 min at 72 °C, and a final extension for 7 min at 72 °C. PCR amplification was carried out on an ABI 9700 GeneAmp PCR system (USA).

DNA sequencing was performed in both directions, initiated from forward and reverse primers, which had been used in an initial PCR reaction. PCR products were purified with Qiagen Purification kit (QIAquick PCR Purification Kit, Germany) and sequenced on ABI PRISM 3130 Genetic Analyser machine (USA). Sequencing data were analysed by using SeqScape 2.5 software, Chromas or equivalent software and compared with the *ATP7B* gene sequence published in Ensembl (ENSG00000123191) by using BioEdit software to detect mutations.

### In silico analysis tools

The consequence of any novel nonsynonomous nucleotide variations that were identified within exons were evaluated with the in silico analysis tools sorting intolerant from tolerant (SIFT) prediction [17], polyphen 2 [18] and mutation taster [19].

## Discussion and conclusions

### Patient 1

**Fig. 1:**
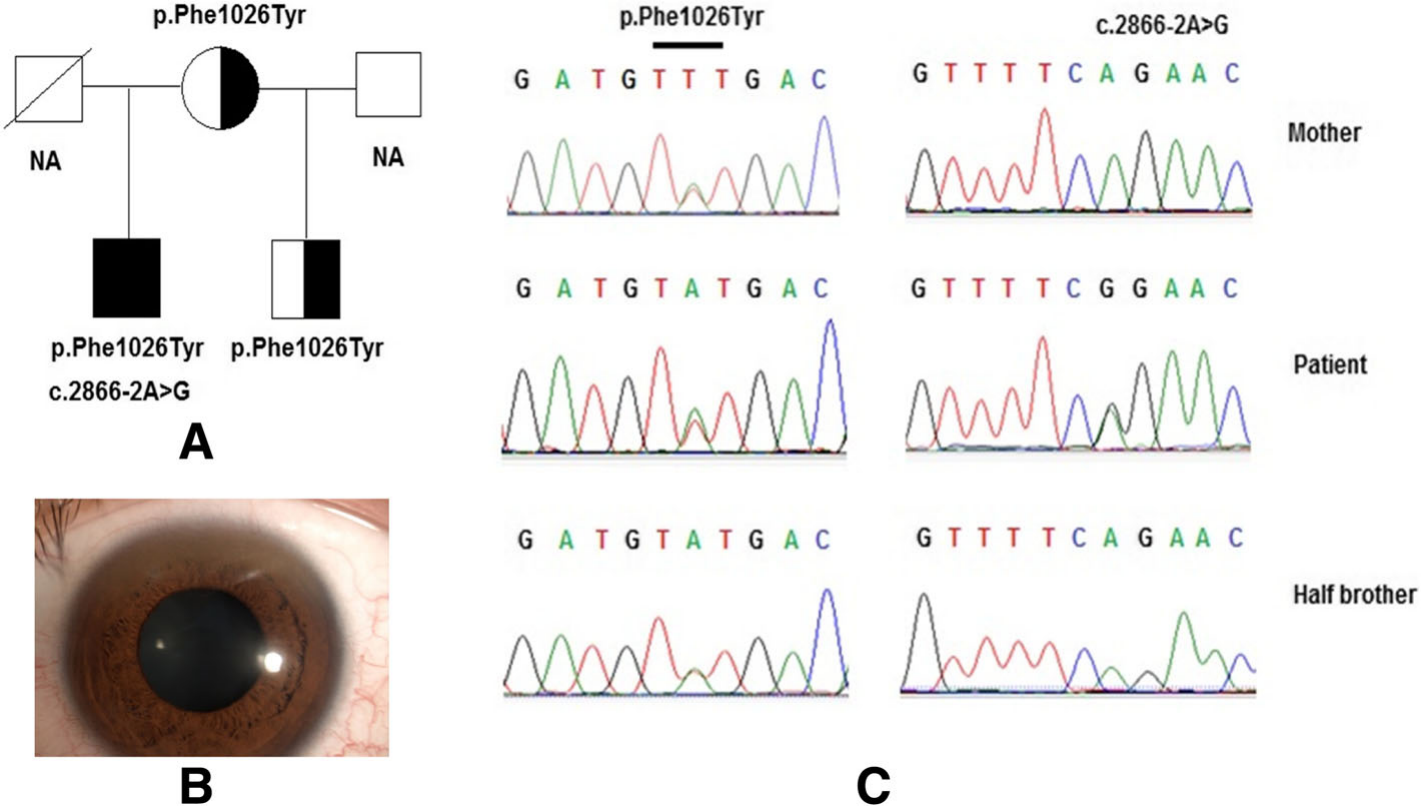
Mutations of the *ATP7B* gene were identified by sequencing in patient 1 and his family. Pedigree of patient’s family (**a**), the Kayser-Fleischer ring around the cornea of patient’s eye (**b**) and mutations (**c**) of *ATP7B* gene were identified, including a compound heterozygous mutation p.Phe1026Tyr and heterozygous mutation c.2866-2A > G

A heterozygous mutation in intron 12 (c.2866-2A > G) and a heterozygous mutation in exon 14 (p.Phe1026Tyr) of the *ATP7B* gene were identified in patient 1. Genetic analysis in his mother showed that she had a heterozygous mutation in exon 14 (p.Phe1026Tyr) but did not have the mutation in intron 12. This result was found the in genotype of the patient’s half brother (Fig. 1). The impact possibility of a missense mutation was evaluated by using bioinformatics tools such as SIFT [17], PolyPhen2 [18] and Mutation Taster [19]. Polyphen-2 analysis for p.Phe1026Tyr substitution in the ATP7B protein of patient 1 indicated a “probably damaging” status with a score of 1.0. Mutation Taster tool predicted the mutation to be “disease causing” with a score of 0.99, and analysis performed by SIFT resulted in a score of 0.01, which indicated that the substitution is “damaging”. These mutations could be the cause of his severe symptoms and early onset of disease. The Human Gene Mutation Database (http://www.hgmd.cf.ac.uk/ac/index.php), and Wilson Disease Mutation Database (http://www.wilsondisease.med.ualberta.ca/search3.asp) were used to determine that the p.Phe1026Tyr change was novel. In addition, c.2866-2A > G was a known mutation with code number CS136099 in The Human Gene Mutation Database (http://www.hgmd.cf.ac.uk/ac/index.php) and its gnomAD frequency was 0.012190% (Table 1).

**Table 1:** GnomAD frequencies for the mutations

Patients	Variants	Exon	Effect	Allele frequencies (%)
Patient 1	c.3077 T > A	14	**p.Phe1026Tyr**	0
	c.2866-2A > G		Splicing	0.012190
Patient 2	c.750_751insG	2	p.His251Alafs*19	0.004730
	c.314C > A	2	p.Ser105*	0.008128
Patient 3	c.2604delC	11	p.Pro868Profs*5	0
	c.314C > A	2	p.Ser105*	0.008128

Bold letters are the novel mutations

Genetic analysis showed that patient 1 had a p.Phe1026Tyr mutation, which was located in exon 14 and the phosphorylation domain (P-domain) of the ATP7B protein. In addition, the patient also carried a mutation in intron 12 (c.2866-2A > G). Patient 1 had the very typically symptoms of the disease such as numbness of hands and feet, vomiting, insomnia, palsy and Kayser–Fleischer rings. Blood and urine biochemical tests manifested that the index of serum ceruloplasmin was very low at 0.0032 mg/dL (Table 2), whereas the concentration of free copper in serum and 24 h urine sample were 3- to 5- fold higher than normal, respectively (serum and urine copper levels of 49 μg/dL and 580 μg, respectively). In addition, a drop in the PT index to 49% indicated a dramatic decline in liver function. The results of in genetic analysis and biochemical tests clearly provided evidence of genotypic and phenotypic correlations in the patient.

**Table 2:** Wilson disease patient clinical data summary

Patient	Sex/Age of onset	Mutations (exon)	Phenotype	Kayser-Freicher rings	Serum ceruloplasmin	Serum free copper	24 h urinary copper	AST	ALT	PT
Normal				–	20–35 mg/dL	< 15 μg/dL	< 60–100 μg	< 40 IU/L	< 40 IU/L	> 70%
Patient 1	Male/8	**p.Phe1026Tyr** (exon 14), c.2866-2A > G (intron 12)	Hepatic, Neurologic	+	0.0032 mg/dL	49 μg/dL	580 μg	26.82 IU/L	20.1 IU/L	49%
Patient 2	Female/8	p.His251Alafs*19 (exon 2), p.Ser105* (exon 2)	Hepatic	+	0.0190 mg/dL	low	150 μg	139.1 IU/L	116.9 IU/L	22%
Patient 3	Male/10	p.Pro868Profs*5 (exon 11), p.Ser105* (exon 2)	Hepatic	+	0.0920 mg/dL	low	3639 μg	129.0 IU/L	55.7 IU/L	17%

Bold letters are the novel mutations

Patient 1 had a very severe and characteristic phenotype of Wilson disease (with clinical features such as numbness of hands and feet, vomiting, insomnia, palsy, liver failure and Kayser–Fleischer rings). This result could be explained by the fact that the patient carried two mutations that were severely affecting the function of protein ATP7B. The mutation p.Phe1026Tyr was located in exon 14 and the phosphorylation domain (P-domain) of the ATP7B protein, which was supposed to have a significant effect on normal protein function. Forbes et al. [20] has given evidence for the effects of ATP7B mutations in severe neuropsychiatric deterioration. Ljubic et al. [21] indicated that mutations in the P-domain interfered with catalytic phosphorylation. In addition, this mutation was compounded by the heterozygous mutation c.2866-2A > G located in the splice site, which can affect mRNA splicing [22].

### Patient 2

**Fig. 2:**
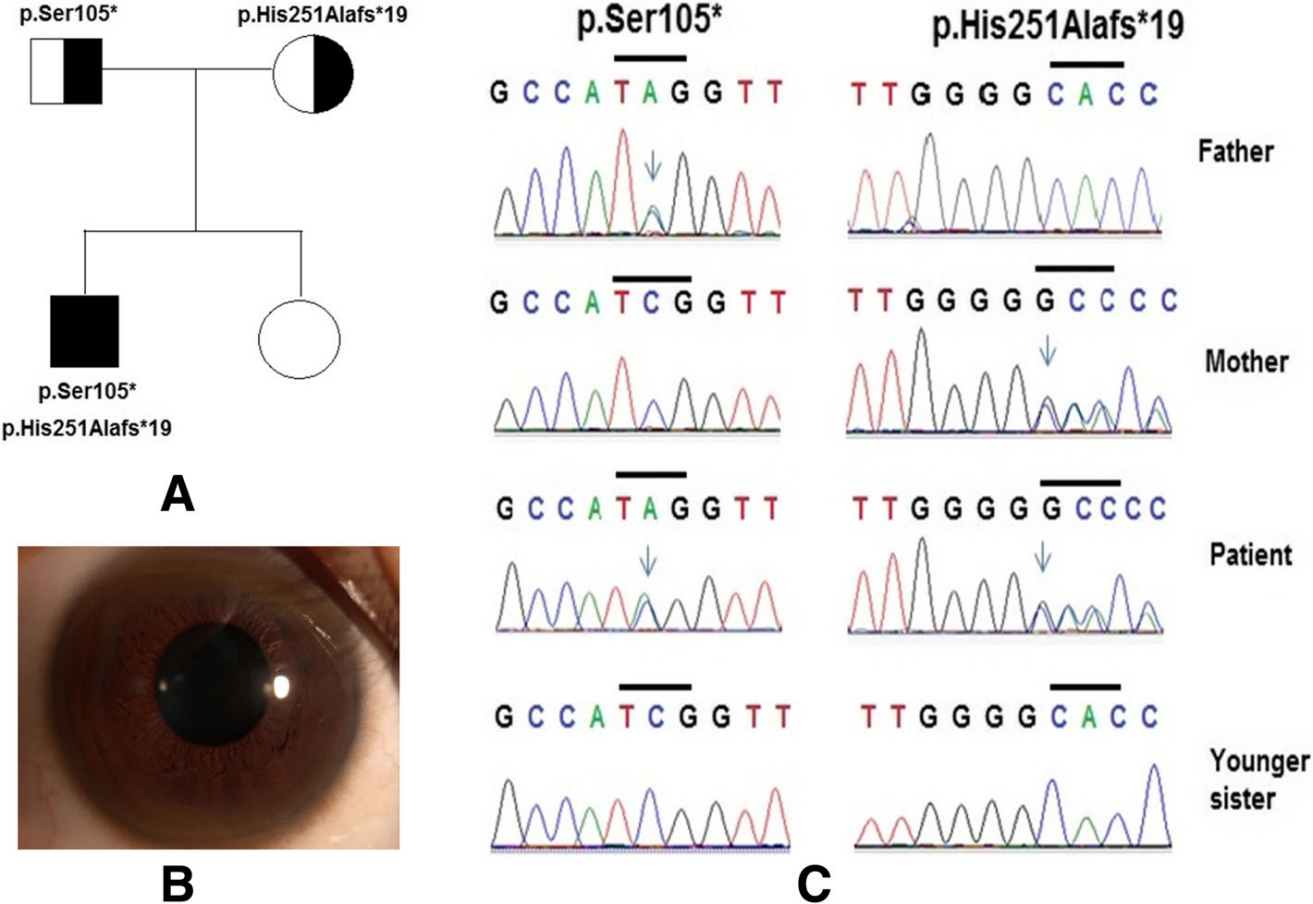
Mutations of the *ATP7B* gene were identified by sequencing in patient 2 and his family. Pedigree of patient’s family (**a**), the Kayser-Fleischer ring around the cornea of patient’s eye (**b**) and mutations (**c**) of *ATP7B* gene were identified, including a compound heterozygous mutation p.Ser105* and heterozygous frameshift mutation p.His251Alafs*19

Two heterozygous mutations in exon 2 (p.Ser105* and p.His251Alafs*19) of the *ATP7B* gene were identified in patient 2. In these mutations, c.314C > A (p.Ser105*) (with gnomAD frequency of 0.008128%) was a known mutation and c.750_751insG (p.His251Alafs*19) (with a gnomAD frequency of 0.004730%) was a novel mutation. Genetic analysis of her family exposed that her father had the heterozygous mutation p.Ser105* and her mother had the heterozygous mutation p.His251Alafs*19 (Fig. 2).

Patient 2 carried two heterozygous mutations, p.Ser105* and p.His251Alafs*19, in exon 2, leading to complete loss of the protein function and early-onset disease. The study by Huong et al. [23] on Vietnamese patients showed that mutations in exon 2 and the p.Ser105* mutation are quite common at a high rate (39.6 and 32.6%, respectively). Exon 2 is the hotspot region of the *ATP7B* gene in many populations, such as Chinese Indian populations, and mutations in exons 2–5 associated with severe phenotypes have been found in the Indian population [24–26]. The investigate of Chen et al. [27] revealed that missense or nonsense mutations caused by single nucleotide variant were very popular (60%), followed by insertions/deletions (26%) and splice-site mutations (9%) in the genetics of Wilson disease. It has previously been suggested that age of onset could potentially be related to the functional activity of *ATP7B* mutants and that truncating mutations in the *ATP7B* gene were associated with an early onset of Wilson disease. Gromadzka et al. [28] noticed that the age of onset was 14 ± 7 years in patients with two severe truncating mutations. In addition, similar results were found by Merle et al. [29], who reported that the age of onset of these patients was in the range of 9–13 years.

### Patient 3

**Fig. 3:**
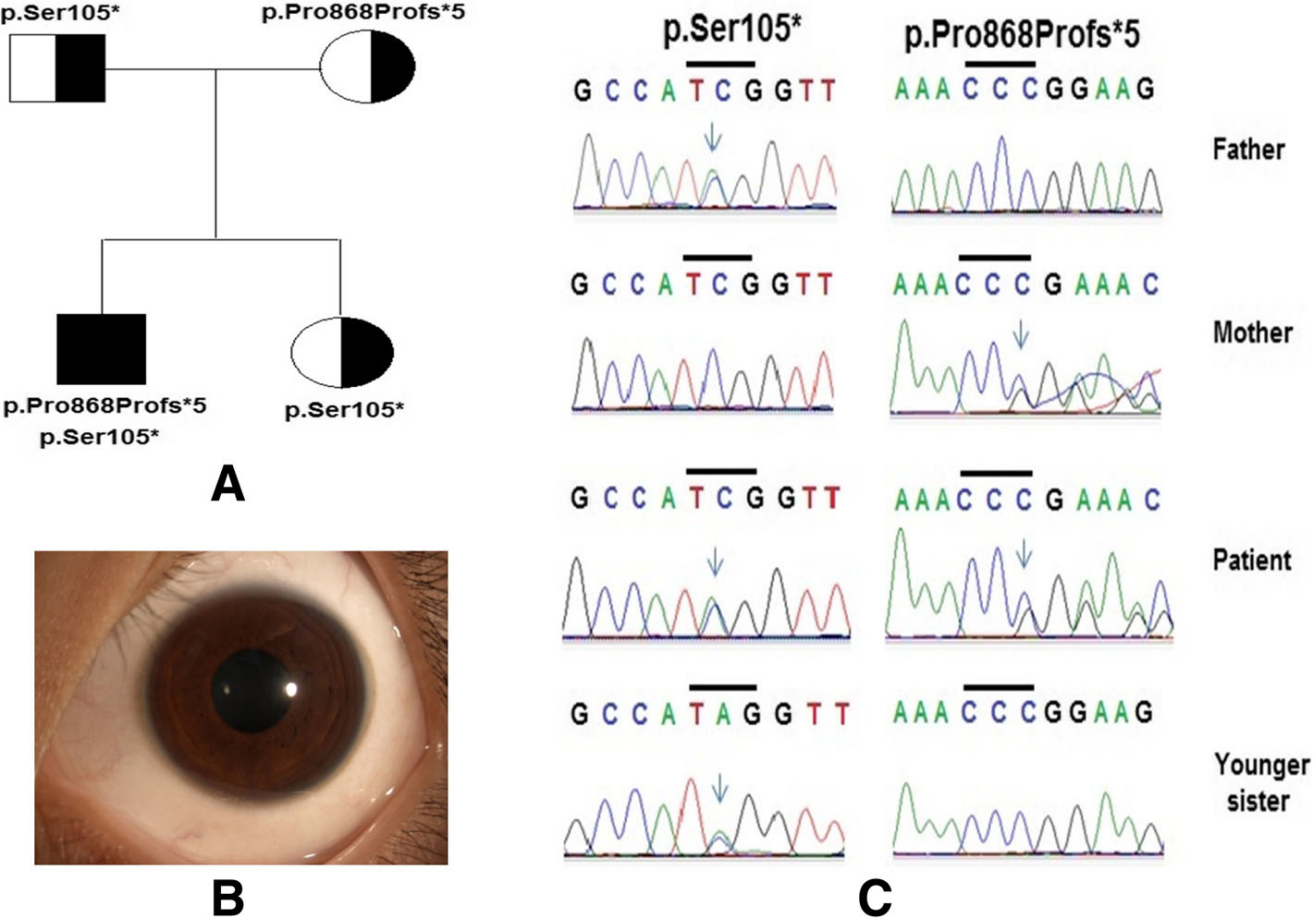
Mutations of the *ATP7B* gene were identified by sequencing in patient 3 and his family. Pedigree of patient’s family (**a**), the Kayser-Fleischer ring around the cornea of patient’s eye (**b**) and mutations (**c**) of *ATP7B* gene were identified, including a compound heterozygous mutation p.Ser105* and heterozygous frameshift mutation p.Pro868Profs*5

In patient 3, a novel heterozygous mutation c.2604delC (p.Pro868Profs*5) was detected in exon 11 of the *ATP7B* gene. Patient 3 also had a heterozygous mutation p.Ser105*. Genetic analysis has shown that the heterozygous mutation p.Ser105* was inherited from his father and that the heterozygous mutation p.Pro868Profs*5 was inherited from his mother (Fig. 3).

Patient 3 carried two severe truncating mutations, which is similar to patient 2. In addition, both patients (patients 2 and 3) had liver damage with early onset. Kalach et al. [30] and Wilson et al. [31] reported cirrhosis in a 3-year-old patient and acute liver failure in a 5-year-old patient, respectively. Yi et al. [32], who established neural and hepatic differentiation platforms of patient-derived induced pluripotent stem cells, showed that liver and brain are the most affected organs. Previous studies have shown that a severe impairment of copper transport resulting in severe liver damage usually occurs in the early stages of Wilson disease [1, 33, 34]. There are rare patients, who present with neurological disease but do not have liver cirrhosis [1].

In both patients 2 and 3, who had the combination of a p.Ser105* mutation and a p.His251Alafs*19 or p.Pro868Profs*5 mutation, the liver was severely damaged with high expression levels of liver enzymes, such as aminotransferases (Table 2). The patients had a high level of aspartate aminotransferase (AST) (139.1 IU/L in patient 2 and 129.0 IU/L in patient 3) and alanine aminotransferase (ALT) (116.9 IU/L in patient 2 and 55.7 IU/L in patient 3). These enzymes are normally predominantly contained within liver cells so that if the liver is injured or damaged, the liver cells spill these enzymes into the blood, raising the AST and ALT enzyme blood levels and signalling liver disease. In these patients, minor reductions in serum ceruloplasmin (with 0.0190 mg/dL and 0.0920 mg/dL, respectively, in patient 2 and 3), serum free copper and the PT index (with 22 and 17%, respectively, for patient 2 and 3) have been observed. Increased urine copper excretion was found in these patients with 150 mg/24 h urine (patient 2) and 3639 mg/24 h urine (patient 3). This fiding is a typical symptom of Wilson disease due to decreased serum binding of copper to ceruloplasmin.

In our study, we identified five nucleotide changes in the *ATP7B* gene in three patients with Wilson disease from three unrelated Vietnamese families. Three of these changes were novel mutations: p.His251Alafs*19 in exon 2, p.Pro868Profs*5 in exon 11, and p.Phe1026Tyr in exon 14. These results provide knowledge on *ATP7B* mutations in patients with Wilson disease and might contribute to the development of effective treatment plans for these patients.

## Additional file

Additional file 1: **Table S1.** List of PCR primers. (DOCX 15.6 kb)

## Abbreviations

ALT: Alanine aminotransferase
AST: Aspartate aminotransferase
ATP7B: ATPase copper transporting beta
DNA: Deoxyribonucleic acid
mRNA: Messenger RNA
PCR: Polymerase chain reaction
PT: Prothromlin time
RNA: Ribonucleic acid
SIFT: Sorting intolerant from tolerant
