# Supplementary material for: Three novel mutations in the ATP7B gene of unrelated Vietnamese patients with Wilson disease
Source: BMC Med Genet. 2018 Jun 18;19:104. doi: 10.1186/s12881-018-0619-4 (PMC6006946; doi:10.1186/s12881-018-0619-4)
Supplement: Supplementary file 1 — Table S1. List of PCR primers. (DOCX 15.6 kb) [file 12881_2018_619_MOESM1_ESM.docx]

**Table 1**. PCR primer sets for amplifying exons of *ATP7B*

| Exon | 5’ primer | 3’ primer | Product size (bp) |
| --- | --- | --- | --- |
| 1 | actttaacaccccgctctc | caagacatccctggagctg | 274 |
| 2a | gttcttctacccttgggatatt | gatggcacatatttcacagt | 392 |
| 2b | cagtcatgtgtgaagtccatt | gtcatttacatggtccctga | 402 |
| 2c | tgagagtcaaagtctcactcag | ctggtacaagaagggtcatact | 406 |
| 2d | ctaggggttcaaagtattcaag | ccttccagttgggagatcat | 311 |
| 2f | acatgcagtaccactctgatt | ctcacctataccaccatcca | 301 |
| 3 | ctgaaacctcttgttctgaaa | agggctactgataaacacagtt | 427 |
| 4 | tttctttgttcggttatattga | aggaaagtgaaacaaacaaaat | 390 |
| 5 | gttacctagactccctggactg | tgaagaattttggttattttca | 357 |
| 6 | ccaatgcatattttaaccaagt | gttcacattacaagggtaaagg | 278 |
| 7 | ggtcttaaactgtgtcctcaga | ataaagtgccatttaaaccaag | 380 |
| 8 | ccctgtgtcgctcattgaactc | tggtgttcagaggaagtgagatttg | 369 |
| 9 | gtctctaacaccacgcttgt | ggattgagagtggtgatcttac | 321 |
| 10 | acagctggcctagaacctgacc | actgtcacttgctcagccccat | 289 |
| 11 | aggtcacatgagtgctggatgg | gggataatctccttcatttaaccaca | 347 |
| 12 | aaatgtggttaaatgaaggaga | gcaagcaaataaaatgtaatga | 364 |
| 13 | ccccctgaaatgtccttatgtga | tgtcttgagtggctctcaggctt | 378 |
| 14 | tggaagcccctccatctgtatt | ttccagaccacacagagaaggct | 345 |
| 15 | cttcaccctgtgtccctgtcct | cttgggtgccttagccatgaac | 385 |
| 16 | ggttaaaaggatattttgctgt | aaattaagagaggaaggctttt | 362 |
| 17 | agaacattgcaagtgtggtat | gaaacacgtggagagaaaag | 357 |
| 18 | gggtaacttgaggtttctgctgc | gcacacagtgaggaaggggtct | 345 |
| 19 | acgtcgtccttatcagagtg | ctttctaaaacgcctctagc | 334 |
| 20 | aggtggagacctcactgg | gtgaatgaatgggaaatgag | 317 |
| 21 | cttcaccaggcttagaaaaa | ctgctcagcttgtggtgagt | 107 |
